# 2-[(*E*)-(Naphthalen-2-yl­imino)­meth­yl]-4-(trifluoro­meth­oxy)phenol

**DOI:** 10.1107/S1600536812009361

**Published:** 2012-03-10

**Authors:** Merve Pekdemir, Zarife Sibel Şahin, Şamil Işık, Ayşen Alaman Ağar, Sema Öztürk Yıldırım, Ray J. Butcher

**Affiliations:** a Department of Physics, Faculty of Arts and Sciences, Ondokuz Mayıs University, Kurupelit, TR-55139 Samsun, Turkey; b Department of Chemistry, Art and Science Faculty, Ondokuz Mayıs University, Kurupelit, TR-55139 Samsun, Turkey; c Department of Physics, Faculty of Sciences, Erciyes University, 38039 Kayseri, Turkey; d Howard University, College of Arts & Sciences, Department of Chemistry, 525 College Street NW, Washington, DC 20059, USA

## Abstract

In the title compound, C_18_H_12_F_3_NO_2_, the planes of the benzene ring and the naphthalene system form a dihedral angle of 47.21 (3)°. The hy­droxy group is involved in an intra­molecular O—H⋯N hydrogen bond. In the crystal, weak C—H⋯O and C—H⋯F inter­actions link the mol­ecules related by translations along the *c* and *a* axes, respectively, into sheets.

## Related literature

For background to photochromic and thermochromic characteristics and tautomerism of Schiff bases, see: Cohen *et al.* (1964 ▶); Hadjoudis *et al.* (1987 ▶). For related structures, see: Gül *et al.* (2007 ▶); Yüce *et al.* (2004 ▶). For classification of hydrogen-bonding patterns, see: Bernstein *et al.* (1995 ▶).
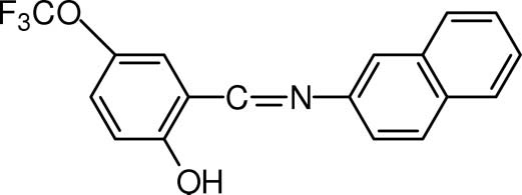


## Experimental

### 

#### Crystal data

C_18_H_12_F_3_NO_2_
*M* *_r_* = 331.29
Monoclinic, 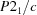

*a* = 17.0813 (10) Å
*b* = 14.1248 (8) Å
*c* = 6.1900 (5) Å
β = 99.669 (6)°
*V* = 1472.25 (17) Å^3^
*Z* = 4
Mo *K*α radiation
μ = 0.12 mm^−1^
*T* = 123 K
0.50 × 0.40 × 0.18 mm

#### Data collection

Oxford Diffraction Gemini-R diffractometer
Absorption correction: analytical [*CrysAlis RED* (Oxford Diffraction, 2007 ▶) based on Clark & Reid (1995 ▶) *T*_min_ = 0.941, *T*_max_ = 0.978
15270 measured reflections
2892 independent reflections
2497 reflections with *I* > 2σ(*I*)
*R*_int_ = 0.050

#### Refinement

*R*[*F* ^2^ > 2σ(*F* ^2^)] = 0.049
*wR*(*F* ^2^) = 0.130
*S* = 1.09
2892 reflections
221 parameters
H atoms treated by a mixture of independent and constrained refinement
Δρ_max_ = 0.31 e Å^−3^
Δρ_min_ = −0.22 e Å^−3^

### 

Data collection: *CrysAlis PRO* (Oxford Diffraction, 2007 ▶); cell refinement: *CrysAlis PRO* (Oxford Diffraction, 2007 ▶); data reduction: *CrysAlis PRO* (Oxford Diffraction, 2007 ▶); program(s) used to solve structure: *SHELXS97* (Sheldrick, 2008 ▶); program(s) used to refine structure: *SHELXL97* (Sheldrick, 2008 ▶); molecular graphics: *ORTEP-3 for Windows* (Farrugia, 1997 ▶); software used to prepare material for publication: *WinGX* (Farrugia, 1999 ▶).

## Supplementary Material

Crystal structure: contains datablock(s) I, global. DOI: 10.1107/S1600536812009361/cv5250sup1.cif

Structure factors: contains datablock(s) I. DOI: 10.1107/S1600536812009361/cv5250Isup2.hkl

Supplementary material file. DOI: 10.1107/S1600536812009361/cv5250Isup3.cml

Additional supplementary materials:  crystallographic information; 3D view; checkCIF report

## Figures and Tables

**Table 1:** Hydrogen-bond geometry (Å, °)

*D*—H⋯*A*	*D*—H	H⋯*A*	*D*⋯*A*	*D*—H⋯*A*
O1—H1⋯N1	0.90 (3)	1.77 (3)	2.5904 (18)	150 (2)
C10—H10⋯O1^i^	0.93	2.57	3.473 (2)	165
C5—H5⋯F2^ii^	0.93	2.57	3.487 (2)	170

Symmetry codes: (i) 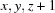; (ii) 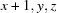.

**Fig. 1.**
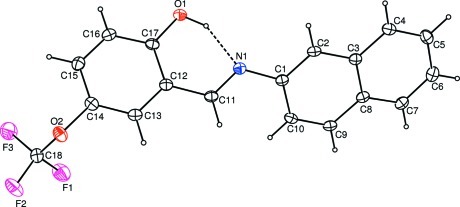
The molecular structure of (I), showing the atom-numbering scheme. Displacement ellipsoids are drawn at the 30% probability. Dashed line denotes hydrogen bond.

**Fig. 2.**
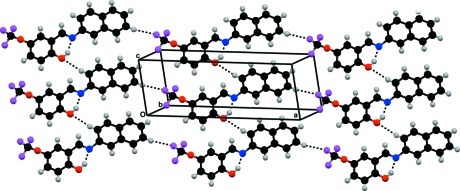
A portion of the crystal packing showing hydrogen bonds as dashed lines.

## supplementary crystallographic information

### Comment

There are two characteristic properties of Schiff bases, *viz*. photochromism and thermochromism (Cohen *et al.*, 1964). These properties result from proton transfer from the hydroxyl O atom to the imine N atom (Hadjoudis *et al.*, 1987). There are two types of intramolecular hydrogen bonds in Schiff bases, which may be stabilized in keto-amine (N—H···O hydrogen bond) or phenol-imine (N···H—O hydrogen bond) tautomeric forms (Hadjoudis *et al.*, 1987). Herewith we present the title compound (I), which exhibits the phenol-imine tautomeric form (Fig. 1).

In (I), the C1—N1 bond length of 1.417 (2) Å agrees with the matching distance in 1-{4-[2-hydroxy-benzylidene)amino]phenyl}ethanone [1.4138 (17) Å; Yüce *et al.*, 2004]. The N1═C11 bond length of 1.284 (2) Å is typical of a double bond, like to the matching bond length in (*E*)-2-[(3-trifluoromenthylphenylimino)methyl]-4-methylphenol [1.280 (2) Å; Gül *et al.*, 2007]. The O1—C17 distance of 1.349 (2) Å is similar to the worth of 1.352 (3) Å in (*E*)-2-[(3-trifluoromenthylphenylimino)methyl]-4-methylphenol (Gül *et al.*, 2007). Fig.1 additionally shows a strong intramolecular hyrogen bond (O1—H1···N1) can be defined as an S(6) motif (Bernstein *et al.*, 1995). The O1—N1 distance of 2.590 (2) Å is comparable to those observed for same hydrogen bonds in 1-{4-[(2-hydroxy-benzylidene)amino]phenyl}ethanone [2.594 (2) Å; Yüce *et al.*, 2004].

The molecules are linked into sheets by a combination of C—H···O and C—H···F interactions (Table 1). The atom C10 in the reference molecule at (*x*, *y*, *z*) acts as a hydrogen-bond donor, *via* H10, to atom O1 in the molecule at (*x*, *y*, *z* + 1), so forming a C(8) chain running parallel to the [001] direction. Similarly, atom C5 in the molecule at (*x*, *y*, *z*) acts as a hydrogen-bond donor, *via* H5, to atom F2 in the molecule at (*x* + 1, *y*, *z*), so forming a C(14) chain running parallel to the [100] direction. The combination of the C(8) and C(14) chains generates a chain edge-fused *R*_5_^5^(36) rings running parallel to the *ac* plane (Fig.2)

### Experimental

The title compound, (I), was prepared by reflux a mixture of a solution containing 2-hydroxy-5-(trifluoromethoxy)benzaldehyde (0.045 g 0.23 mmol) in 20 ml e thanol and a solution containing 2-Naphthyamine (0.033 g 0.23 mmol) in 20 ml e thanol. The reaction mixture was stirred for 1 hunder reflux. The crystals of (I) suitable for X-ray analysis were obtained from ethylalcohol by slow evaporation (yield % 68; m.p.369–371 K).

### Refinement

The H1 atom was located in a difference map, and isotropically refined with restraint of O—H=0.82 (2) Å. All other H atoms were placed in calculated positions and constrained to ride on their parents atoms, with C—H=0.93 Å and *U*_iso_(H)=1.2*U*_eq_(C).

### Crystal data

**Table:**

C_18_H_12_F_3_NO_2_	*F*(000) = 680
*M**_r_* = 331.29	*D*_x_ = 1.495 Mg m^−^^3^
Monoclinic, *P*2_1_/*c*	Mo *K*α radiation, λ = 0.71073 Å
Hall symbol: -P 2ybc	Cell parameters from 5076 reflections
*a* = 17.0813 (10) Å	θ = 3.1–34.9°
*b* = 14.1248 (8) Å	µ = 0.12 mm^−^^1^
*c* = 6.1900 (5) Å	*T* = 123 K
β = 99.669 (6)°	Plate, yellow
*V* = 1472.25 (17) Å^3^	0.50 × 0.40 × 0.18 mm
*Z* = 4	

### Data collection

**Table:**

Oxford Diffraction Gemini-R diffractometer	2892 independent reflections
Radiation source: Enhance (Mo) X-ray Source	2497 reflections with *I* > 2σ(*I*)
Graphite monochromator	*R*_int_ = 0.050
Detector resolution: 10.5081 pixels mm^-1^	θ_max_ = 26.0°, θ_min_ = 3.1°
ω scans	*h* = −20→21
Absorption correction: analytical [*CrysAlis RED* (Oxford Diffraction, 2007) based on Clark & Reid (1995)	*k* = −17→17
*T*_min_ = 0.941, *T*_max_ = 0.978	*l* = −7→6
15270 measured reflections	

### Refinement

**Table:**

Refinement on *F*^2^	Primary atom site location: structure-invariant direct methods
Least-squares matrix: full	Secondary atom site location: difference Fourier map
*R*[*F*^2^ > 2σ(*F*^2^)] = 0.049	Hydrogen site location: inferred from neighbouring sites
*wR*(*F*^2^) = 0.130	H atoms treated by a mixture of independent and constrained refinement
*S* = 1.09	*w* = 1/[σ^2^(*F*_o_^2^) + (0.062*P*)^2^ + 0.5277*P*] where *P* = (*F*_o_^2^ + 2*F*_c_^2^)/3
2892 reflections	(Δ/σ)_max_ < 0.001
221 parameters	Δρ_max_ = 0.31 e Å^−^^3^
0 restraints	Δρ_min_ = −0.22 e Å^−^^3^

### Special details

**Table:**

Geometry. All e.s.d.'s (except the e.s.d. in the dihedral angle between two l.s. planes) are estimated using the full covariance matrix. The cell e.s.d.'s are taken into account individually in the estimation of e.s.d.'s in distances, angles and torsion angles; correlations between e.s.d.'s in cell parameters are only used when they are defined by crystal symmetry. An approximate (isotropic) treatment of cell e.s.d.'s is used for estimating e.s.d.'s involving l.s. planes.
Refinement. Refinement of *F*^2^ against ALL reflections. The weighted *R*-factor *wR* and goodness of fit *S* are based on *F*^2^, conventional *R*-factors *R* are based on *F*, with *F* set to zero for negative *F*^2^. The threshold expression of *F*^2^ > σ(*F*^2^) is used only for calculating *R*-factors(gt) *etc*. and is not relevant to the choice of reflections for refinement. *R*-factors based on *F*^2^ are statistically about twice as large as those based on *F*, and *R*- factors based on ALL data will be even larger.

### Fractional atomic coordinates and isotropic or equivalent isotropic displacement parameters (Å^2^)

**Table:**

	*x*	*y*	*z*	*U*_iso_*/*U*_eq_	
C1	0.52707 (9)	0.88417 (11)	0.3403 (3)	0.0225 (4)	
C2	0.59465 (10)	0.90836 (11)	0.2604 (3)	0.0225 (3)	
H2	0.5903	0.9338	0.1203	0.027*	
C3	0.67071 (9)	0.89512 (10)	0.3876 (3)	0.0222 (3)	
C4	0.74184 (10)	0.91509 (11)	0.3061 (3)	0.0268 (4)	
H4	0.7390	0.9413	0.1672	0.032*	
C5	0.81420 (10)	0.89635 (12)	0.4287 (3)	0.0313 (4)	
H5	0.8601	0.9103	0.3729	0.038*	
C6	0.82014 (11)	0.85608 (12)	0.6391 (3)	0.0320 (4)	
H6	0.8698	0.8424	0.7201	0.038*	
C7	0.75296 (10)	0.83705 (11)	0.7244 (3)	0.0275 (4)	
H7	0.7573	0.8114	0.8642	0.033*	
C8	0.67680 (10)	0.85604 (11)	0.6021 (3)	0.0232 (4)	
C9	0.60574 (10)	0.83457 (11)	0.6822 (3)	0.0246 (4)	
H9	0.6089	0.8110	0.8237	0.029*	
C10	0.53290 (10)	0.84767 (11)	0.5564 (3)	0.0244 (4)	
H10	0.4871	0.8327	0.6121	0.029*	
C11	0.38798 (10)	0.90319 (11)	0.2662 (3)	0.0241 (4)	
H11	0.3894	0.9215	0.4112	0.029*	
C12	0.31186 (10)	0.89349 (11)	0.1221 (3)	0.0236 (4)	
C13	0.24206 (10)	0.92206 (11)	0.1940 (3)	0.0246 (4)	
H13	0.2443	0.9475	0.3334	0.030*	
C14	0.17025 (10)	0.91238 (11)	0.0580 (3)	0.0265 (4)	
C15	0.16428 (10)	0.87238 (12)	−0.1487 (3)	0.0285 (4)	
H15	0.1150	0.8661	−0.2381	0.034*	
C16	0.23260 (10)	0.84183 (12)	−0.2206 (3)	0.0279 (4)	
H16	0.2290	0.8130	−0.3570	0.034*	
C17	0.30663 (10)	0.85398 (11)	−0.0901 (3)	0.0248 (4)	
C18	0.05121 (10)	0.89134 (14)	0.1960 (3)	0.0335 (4)	
F1	0.08590 (7)	0.83013 (9)	0.3436 (2)	0.0551 (4)	
F2	−0.00045 (7)	0.94076 (10)	0.2849 (2)	0.0537 (4)	
F3	0.01029 (7)	0.84060 (11)	0.0357 (2)	0.0614 (4)	
N1	0.45326 (8)	0.88684 (9)	0.1961 (2)	0.0241 (3)	
O1	0.37203 (7)	0.82747 (9)	−0.1701 (2)	0.0301 (3)	
O2	0.10202 (7)	0.95092 (8)	0.1277 (2)	0.0328 (3)	
H1	0.4140 (16)	0.8422 (18)	−0.067 (5)	0.066 (8)*	

### Atomic displacement parameters (Å^2^)

**Table:**

	*U*^11^	*U*^22^	*U*^33^	*U*^12^	*U*^13^	*U*^23^
C1	0.0276 (8)	0.0185 (8)	0.0218 (8)	0.0017 (6)	0.0051 (7)	−0.0016 (6)
C2	0.0325 (9)	0.0176 (7)	0.0184 (8)	0.0003 (6)	0.0072 (7)	0.0002 (6)
C3	0.0282 (8)	0.0162 (7)	0.0228 (8)	0.0003 (6)	0.0060 (7)	−0.0030 (6)
C4	0.0327 (9)	0.0225 (8)	0.0268 (9)	−0.0017 (7)	0.0095 (7)	−0.0011 (7)
C5	0.0279 (9)	0.0273 (9)	0.0402 (10)	−0.0031 (7)	0.0102 (8)	−0.0038 (8)
C6	0.0299 (9)	0.0266 (9)	0.0370 (10)	0.0026 (7)	−0.0018 (8)	−0.0041 (8)
C7	0.0349 (9)	0.0205 (8)	0.0256 (9)	0.0014 (6)	0.0013 (7)	−0.0025 (6)
C8	0.0317 (9)	0.0158 (7)	0.0220 (8)	0.0005 (6)	0.0043 (7)	−0.0026 (6)
C9	0.0345 (9)	0.0215 (8)	0.0185 (8)	0.0009 (6)	0.0070 (7)	0.0011 (6)
C10	0.0282 (8)	0.0223 (8)	0.0253 (9)	−0.0004 (6)	0.0118 (7)	−0.0016 (6)
C11	0.0304 (9)	0.0207 (8)	0.0222 (8)	0.0005 (6)	0.0069 (7)	−0.0012 (6)
C12	0.0292 (8)	0.0191 (8)	0.0231 (8)	−0.0001 (6)	0.0063 (7)	0.0026 (6)
C13	0.0323 (9)	0.0191 (8)	0.0235 (8)	0.0006 (6)	0.0076 (7)	−0.0003 (6)
C14	0.0277 (8)	0.0224 (8)	0.0303 (9)	0.0020 (6)	0.0080 (7)	0.0031 (7)
C15	0.0291 (9)	0.0270 (8)	0.0282 (9)	−0.0026 (7)	0.0014 (7)	0.0036 (7)
C16	0.0350 (9)	0.0264 (8)	0.0221 (8)	−0.0016 (7)	0.0041 (7)	0.0003 (7)
C17	0.0303 (9)	0.0213 (8)	0.0245 (9)	−0.0010 (6)	0.0092 (7)	0.0010 (6)
C18	0.0257 (9)	0.0413 (11)	0.0330 (10)	0.0012 (7)	0.0035 (8)	−0.0022 (8)
F1	0.0503 (7)	0.0630 (8)	0.0554 (8)	0.0110 (6)	0.0188 (6)	0.0264 (6)
F2	0.0386 (7)	0.0587 (8)	0.0701 (9)	0.0065 (5)	0.0274 (6)	−0.0067 (6)
F3	0.0440 (7)	0.0829 (10)	0.0581 (8)	−0.0267 (7)	0.0108 (6)	−0.0259 (7)
N1	0.0275 (7)	0.0215 (7)	0.0236 (7)	−0.0009 (5)	0.0052 (6)	0.0006 (5)
O1	0.0299 (7)	0.0375 (7)	0.0244 (6)	0.0001 (5)	0.0085 (5)	−0.0058 (5)
O2	0.0283 (6)	0.0289 (7)	0.0428 (8)	0.0036 (5)	0.0103 (5)	−0.0008 (5)

### Geometric parameters (Å, º)

**Table:**

C1—C2	1.373 (2)	C11—N1	1.284 (2)
C1—N1	1.417 (2)	C11—C12	1.454 (2)
C1—C10	1.421 (2)	C11—H11	0.9300
C2—C3	1.414 (2)	C12—C13	1.400 (2)
C2—H2	0.9300	C12—C17	1.416 (2)
C3—C4	1.420 (2)	C13—C14	1.373 (2)
C3—C8	1.425 (2)	C13—H13	0.9300
C4—C5	1.363 (2)	C14—C15	1.387 (2)
C4—H4	0.9300	C14—O2	1.4176 (19)
C5—C6	1.409 (3)	C15—C16	1.386 (2)
C5—H5	0.9300	C15—H15	0.9300
C6—C7	1.368 (3)	C16—C17	1.392 (2)
C6—H6	0.9300	C16—H16	0.9300
C7—C8	1.416 (2)	C17—O1	1.349 (2)
C7—H7	0.9300	C18—F2	1.316 (2)
C8—C9	1.419 (2)	C18—F1	1.324 (2)
C9—C10	1.365 (2)	C18—F3	1.324 (2)
C9—H9	0.9300	C18—O2	1.328 (2)
C10—H10	0.9300	O1—H1	0.90 (3)
			
C2—C1—N1	118.63 (14)	N1—C11—C12	120.90 (15)
C2—C1—C10	119.94 (15)	N1—C11—H11	119.5
N1—C1—C10	121.10 (14)	C12—C11—H11	119.5
C1—C2—C3	121.05 (15)	C13—C12—C17	118.92 (15)
C1—C2—H2	119.5	C13—C12—C11	119.96 (15)
C3—C2—H2	119.5	C17—C12—C11	121.11 (15)
C2—C3—C4	122.47 (15)	C14—C13—C12	119.83 (15)
C2—C3—C8	119.10 (14)	C14—C13—H13	120.1
C4—C3—C8	118.35 (15)	C12—C13—H13	120.1
C5—C4—C3	120.90 (16)	C13—C14—C15	121.73 (15)
C5—C4—H4	119.6	C13—C14—O2	118.08 (15)
C3—C4—H4	119.6	C15—C14—O2	120.04 (15)
C4—C5—C6	120.71 (16)	C16—C15—C14	119.18 (16)
C4—C5—H5	119.6	C16—C15—H15	120.4
C6—C5—H5	119.6	C14—C15—H15	120.4
C7—C6—C5	120.07 (16)	C15—C16—C17	120.49 (16)
C7—C6—H6	120.0	C15—C16—H16	119.8
C5—C6—H6	120.0	C17—C16—H16	119.8
C6—C7—C8	120.76 (16)	O1—C17—C16	118.72 (15)
C6—C7—H7	119.6	O1—C17—C12	121.51 (15)
C8—C7—H7	119.6	C16—C17—C12	119.77 (15)
C7—C8—C9	122.39 (15)	F2—C18—F1	108.15 (15)
C7—C8—C3	119.20 (15)	F2—C18—F3	107.09 (14)
C9—C8—C3	118.38 (15)	F1—C18—F3	106.41 (17)
C10—C9—C8	121.55 (15)	F2—C18—O2	108.49 (16)
C10—C9—H9	119.2	F1—C18—O2	113.14 (14)
C8—C9—H9	119.2	F3—C18—O2	113.28 (15)
C9—C10—C1	119.93 (15)	C11—N1—C1	121.55 (14)
C9—C10—H10	120.0	C17—O1—H1	106.7 (17)
C1—C10—H10	120.0	C18—O2—C14	117.93 (13)
			
N1—C1—C2—C3	−171.57 (13)	C17—C12—C13—C14	−0.7 (2)
C10—C1—C2—C3	1.8 (2)	C11—C12—C13—C14	−179.68 (14)
C1—C2—C3—C4	176.90 (14)	C12—C13—C14—C15	1.8 (2)
C1—C2—C3—C8	0.0 (2)	C12—C13—C14—O2	−173.65 (14)
C2—C3—C4—C5	−176.17 (15)	C13—C14—C15—C16	−0.4 (2)
C8—C3—C4—C5	0.8 (2)	O2—C14—C15—C16	174.97 (14)
C3—C4—C5—C6	0.4 (3)	C14—C15—C16—C17	−2.1 (2)
C4—C5—C6—C7	−1.3 (3)	C15—C16—C17—O1	−176.85 (14)
C5—C6—C7—C8	1.0 (3)	C15—C16—C17—C12	3.2 (2)
C6—C7—C8—C9	178.00 (15)	C13—C12—C17—O1	178.27 (14)
C6—C7—C8—C3	0.2 (2)	C11—C12—C17—O1	−2.7 (2)
C2—C3—C8—C7	175.98 (14)	C13—C12—C17—C16	−1.8 (2)
C4—C3—C8—C7	−1.1 (2)	C11—C12—C17—C16	177.22 (14)
C2—C3—C8—C9	−1.9 (2)	C12—C11—N1—C1	−171.85 (13)
C4—C3—C8—C9	−178.95 (14)	C2—C1—N1—C11	−151.42 (15)
C7—C8—C9—C10	−175.68 (15)	C10—C1—N1—C11	35.3 (2)
C3—C8—C9—C10	2.1 (2)	F2—C18—O2—C14	170.19 (14)
C8—C9—C10—C1	−0.4 (2)	F1—C18—O2—C14	50.2 (2)
C2—C1—C10—C9	−1.6 (2)	F3—C18—O2—C14	−71.0 (2)
N1—C1—C10—C9	171.60 (14)	C13—C14—O2—C18	−105.77 (18)
N1—C11—C12—C13	−172.63 (14)	C15—C14—O2—C18	78.7 (2)
N1—C11—C12—C17	8.4 (2)		

### Hydrogen-bond geometry (Å, º)

**Table:**

*D*—H···*A*	*D*—H	H···*A*	*D*···*A*	*D*—H···*A*
O1—H1···N1	0.90 (3)	1.77 (3)	2.5904 (18)	150 (2)
C10—H10···O1^i^	0.93	2.57	3.473 (2)	165
C5—H5···F2^ii^	0.93	2.57	3.487 (2)	170

Symmetry codes: (i) *x*, *y*, *z*+1; (ii) *x*+1, *y*, *z*.
